# First case of a renal cyst infection caused by *Desulfovibrio*: a case report and literature review

**DOI:** 10.1186/s12882-022-02803-w

**Published:** 2022-05-23

**Authors:** Yoshiki Okamoto, Yoei Miyabe, Momoko Seki, Yusuke Ushio, Keisuke Sato, Eri Kasama, Kenichi Akiyama, Kazunori Karasawa, Keiko Uchida, Ken Kikuchi, Kosaku Nitta, Takahito Moriyama, Junichi Hoshino

**Affiliations:** 1 Department of Nephrology, Tokyo Women’s Medical University, 8-1 Kawada-Cho, Shinjuku-ku, Tokyo, 1628666 Japan; 2 Department of Infectious Diseases, Tokyo Women’s Medical University, 8-1 Kawada-Cho, Shinjuku-ku, Tokyo, 1628666 Japan

**Keywords:** *Desulfovibrio* species, *Desulfovibrio fairfieldensis*, Renal cyst infection, Haemodialysis, Case report

## Abstract

**Background:**

Genus *Desulfovibrio* species is a sulphate-reducing anaerobic gram-negative rod that resides in the human oral cavity and intestinal tract. It was reported as the causative pathogen of bacteraemia and abdominal infections, but not renal cyst infection, and *Desulfovibrio fairfieldensis* has higher pathogenicity than other *Desulfovibrio* species.

**Case presentation:**

A 63-year-old man was on haemodialysis for end-stage renal failure due to autosomal dominant polycystic kidney disease. On admission, he had a persistent high-grade fever, right lumbar back pain, and elevated C-reactive protein levels. His blood and urine cultures were negative. He received ciprofloxacin and meropenem; however, there was no clinical improvement. Contrast-enhanced computed tomography and plain magnetic resonance imaging revealed a haemorrhagic cyst at the upper pole of the right kidney. The lesion was drained. Although the drainage fluid culture was negative, *D. fairfieldensis* was detected in a renal cyst using a polymerase chain reaction. After the renal cyst drainage, he was treated with oral metronidazole and improved without any relapse.

**Conclusions:**

To the best of our knowledge, this is the first reported case of a renal cyst infection with *Desulfovibrio* species*. D. fairfieldensis* is difficult to detect, and polymerase chain reaction tests can detect this bacterium and ensure better management for a successful recovery.

## Background

Genus *Desulfovibri*o is an anaerobic gram-negative rod and a type of sulphate-reducing bacteria belonging to more than 30 species residing in the human oral cavity, intestinal tract, and nature, including soil, sewage, and brackish water [1]. *Desulfovibrio fairfieldensis* has higher pathogenicity and more antimicrobial resistance than other *Desulfovibrio* species [1–4]. It may be the causative pathogen of bacteraemia and abdominal infections, such as abscesses and cholecystitis [1]. There are several reports of infections such as brain abscesses, meningitis, intra-abdominal abscesses, and bacteraemia caused by *Desulfovibrio* species [1–3, 5–7], but not renal cyst infection. Here, we report a case of renal cyst infection caused by *D. fairfieldensis;* this is the first such report.

## Case presentation

**Fig. 1:**
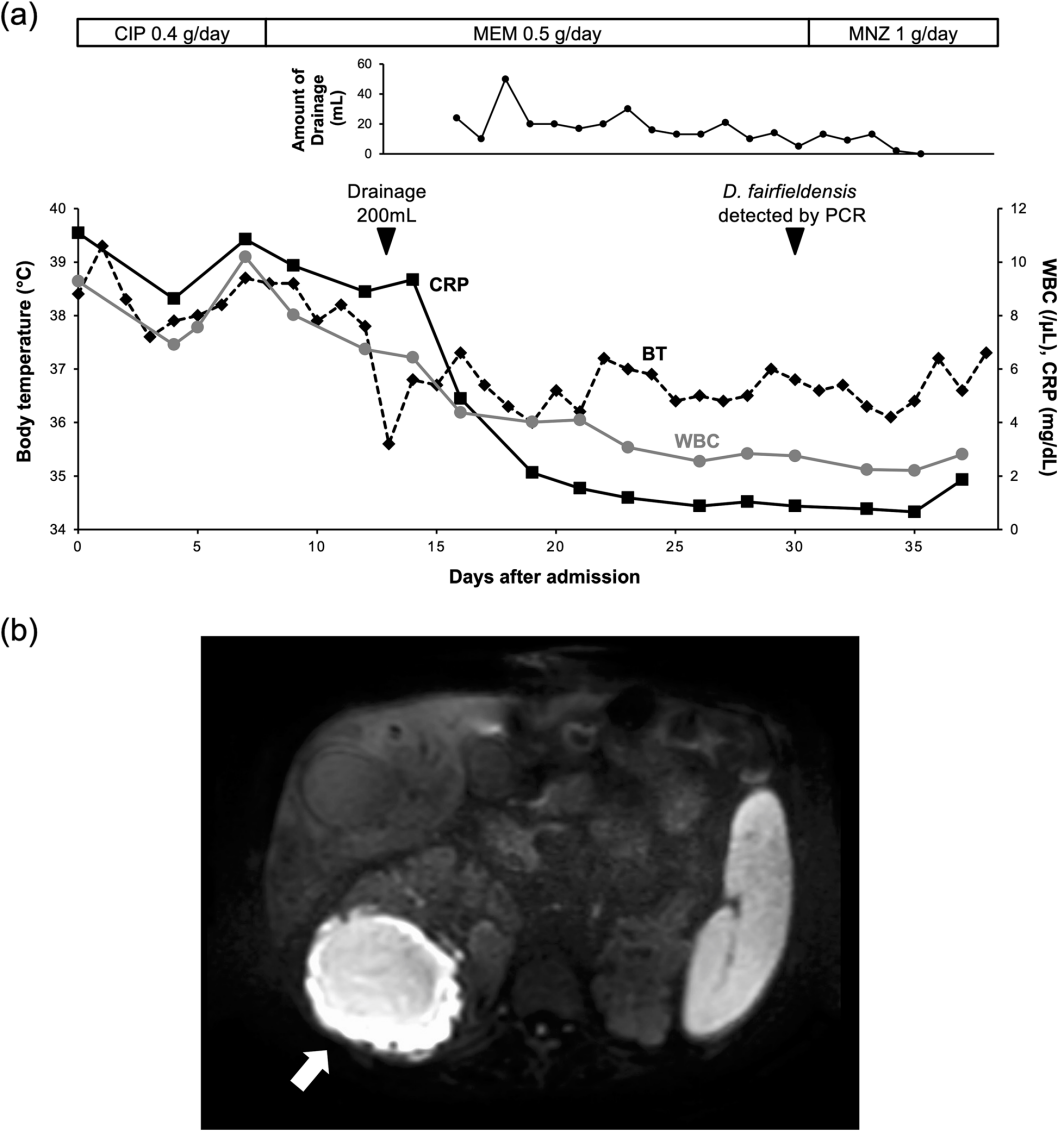
**a** Chart of patient’s clinical course after admission. The lower graph shows the patient’s body temperature, white blood cell count, and C-reactive protein levels during hospitalisation. Renal cyst drainage was performed on Day 13, and the fluid drained initially was 200 mL. A PCR test performed on Day 30 revealed that the causative bacteria was *Desulfovibrio fairfieldensis*. The middle graph shows the volume of fluid drained. The drained fluid volume could not be measured for two days after the initial drainage. The upper bar shows the antibacterial drug administered, the dose, and the timing of switching; BT, body temperature; CIP, ciprofloxacin; CRP, C-reactive protein; MEM, meropenem; MNZ, oral metronidazole; PCR, polymerase chain reaction; WBC, white blood cell count. **b** Diffusion-weighted imaging of plain abdominal magnetic resonance imaging (MRI) on Day 9 of admission. White arrow: a renal haemorrhagic cyst

A 63-year-old man, who was a glass craftsman and a sewer cleaner, on haemodialysis for 19 years due to autosomal dominant polycystic kidney disease (ADPKD), was referred by his family doctor for suspicion of renal cyst infection after presenting with a persistent fever of approximately 38 °C, right lumbar back pain, and elevated C-reactive protein (CRP) levels for the past 14 days. Although he had received intravenous ceftriaxone for two days and meropenem and levofloxacin for 12 days, he displayed no clinical improvement. On admission, he had a fever of 38.4 °C and negative blood and urine cultures (Fig. 1a). His blood tests revealed leucocytosis (9280/μL), thrombocytopenia (77000/μL), elevated CRP levels (11.09 mg/dL), and elevated procalcitonin levels (0.94 ng/mL). Plain computed tomography (CT) revealed a right renal cyst infection. Although treatment with intravenous ciprofloxacin (0.4 g/day) had been started, his clinical findings did not improve. Therefore, his treatment was changed to meropenem (0.5 g/day) on Day 9 to cover extended-spectrum β-lactamase-producing bacteria since meropenem had been reported to provide poor penetration into infected cysts but clinical improvement [8]. Contrast-enhanced CT and plain magnetic resonance imaging (MRI) were also performed (Fig. 1b). They revealed a haemorrhagic cyst at the upper pole of the right kidney, which was suspected to be the cause of the infection; percutaneous drainage of the renal cyst was performed on Day 13, and 200 mL of fluid was drained. The subsequent drainage volume was approximately 20 mL daily for 1 week. After drainage, the patient’s body temperature reduced to approximately 36.7 °C. In addition, the leucocytosis, thrombocytopenia, elevated CRP, and procalcitonin levels were resolved. The drainage fluid culture was negative for bacteria, including anaerobes and fungi. Therefore, a polymerase chain reaction (PCR) test of 16S rDNA using 27FN (AGAGTTTGATCMTGGCTCAG) and 1525R (AAAGGAGGTGATCCAGCC) primers was performed for purified DNA from the drainage fluid. On Day 30, it turned out that the obtained sequences were 99.7% identical (1500/1505 bp) to that of *D. fairfieldensis* ATCC 700045^T^(U42221). Therefore, on Day 31, his treatment was changed to oral metronidazole (1 g/day). The volume of drained fluid decreased to 0–2 mL on Day 34, and contrast-enhanced CT performed on Day 35 showed shrinkage of the renal cysts. His clinical findings normalised, and the drainage tube was removed on Day 36. The Japanese guidelines for treating renal cyst infection in patients with ADPKD recommend a treatment period of at least 4 weeks with antimicrobial agents [9]. Therefore, on Day 38, he was discharged and asked to continue oral metronidazole for 4 weeks. After that, there was no relapse of the infection.

## Discussion and conclusions

Genus *Desulfovibrio* was first described in 1895 [10], and a human infection (bacteraemia associated with cholecystitis) with *D. desulfuricans* was first reported in 1987 [11]. However, it was later considered to be *D. fairfieldensis* in 2005 because the strain was positive for catalase and nitrate. Optical and electron micrographs of *D. fairfieldensis* were published in 1996 and 1997 [6, 12], and the first human infection with *D. fairfieldensis* was reported in Fairfield, Australia; it presented as a liver abscess [6]. Subsequently, we searched PubMed and Google scholar and 71 reported human cases of infection with *Desulfovibrio* species, including *D. desulfuricans*, *D. fairfieldensis*, *D. piger*, and *D. legalli*, were found in 26 articles (Table 1). *D. fairfieldensis* has been isolated from several sites of infection, including blood [2–4, 7, 12], peritoneal fluid [4], periodontal pockets [29, 30], the pelvis and colon [4], liver abscesses [6], and urine [5]. This report describes the first case of renal cyst infection caused by the genus *Desulfovibrio*. When our case is added to those previously reported, *D. fairfieldensis* infection is the most common (26 cases, 36%), followed by *D. desulfuricans* (24 cases, 33%), with bacteraemia and intra-abdominal infection being the commonest presentations (Table 2).

**Table 1:** Characteristics of 72 cases infected with *Desulfovibrio* species in 27 articles

Case no.	Age (yrs)	Sex	Infection	Source	Genus/species	Co-isolated/co-infected organism(s)	Identification	Time for positive incubation	Antibiotic susceptibility (Susceptible)	Antibiotic therapy	Outcome	Ref.
1	39	M	Sinusitis, gingivitis, brain abscess	Pus	*D. desulfuricans*	*Streptococcus constellatus, Capnocytophaga ochracea, Cubacterium exiguum*	Biochemical	10 days	AMC, IPM, MNZ	CTX, FOF, ONZ, PIP, PEF	Survived	[3, 13]
2	3	F	Appendix abscess	Pus	*D. desulfuricans*	*B. merdae, E. lentum, E. coli, Enterococcus* sp.	16S rDNA	unknown	unknown	unknown	Survived	[3]
3	61	F	Abdominal wall abscess, peritonitis	Pus	*D. desulfuricans*	*B. fragilis, E. lentum, Clostridium* sp.*, E. coli, Enterobacter cloacae, Enterococcus* sp.	16S rDNA	unknown	unknown	unknown	Survived	
				Blood	*D. desulfuricans*	*E. coli, Enterobacter cloacae*	16S rDNA	unknown	unknown			
4	80	M	Peritonitis	Peritoneal fluid	*D. desulfuricans*	unknown	16S rDNA	unknown	unknown	unknown	unknown	[14]
5	64	M	Bacteraemia	Blood	*D. desulfuricans*	None	16S rDNA	6 days	LVX, MXF, GAT, MNZ, CLI, IPM, ETP, DOX	DOX	Survived	[1]
6–8	unknown	unknown	unknown	unknown	*D. desulfuricans*	unknown	unknown	unknown	unknown	unknown	unknown	[4]
9	86	F	Bacteraemia, sacral decubitus ulcer	Blood	*D. desulfuricans*	*E. lenta*	16S rDNA	5 days	AMX, AMC, CLI, IPM, MNZ	CXM, AMX	Survived	[15]
10	60	M	Bacteraemia	Blood	*D. desulfuricans*	None	16S rDNA	8 days	unknown	CRO, ERY, PIP	Survived	[16]
11	69	F	Bacteraemia, ulcerative colitis	Blood	*D. desulfuricans*	*Cytomegalovirus*	16S rDNA	7 days	CLI, MNZ, ERY, AMC, MEM	PIP, CLI	Survived	[17]
12	87	M	Bacteraemia, colitis	Blood	*D. desulfuricans*	None	16S rDNA	12 days	SAM, TZP, AMC, FEP, MEM	SAM, CFZ, CAZ, CZO	Survived	[18]
13	69	M	Bacteraemia	Blood	*D. desulfuricans*	None	16S rDNA	5 days	IPM, MNZ	OFX, TZP	Survived	[19]
14	66	F	Hydronephrosis, suspected colon-ureteral/vesical fistula	Urine from percutaneous nephrostomy	*D. desulfuricans*	Anaerobic Gram-positive bacilli, anaerobic Gram-positive cocci, *Streptococcus agalactiae, Actinobaculum schaalii, Propionimicrobium* spp.	16S rDNA	unknown	CLI, MNZ, PEN	unknown	Died, secondary to herpes encephalitis	[20]
15	76	M	Bacteraemia, diverticulitis	Blood	*D. desulfuricans*	None	16S rDNA	3 days	MNZ	unknown	Survived	
16	60	M	Colonic rupture	Spine tissue	*D. desulfuricans*	*Mobiluncus curtisii, Candida albicans, Clostridium clostridioforme*	16S rDNA	unknown	CLI, MNZ, PEN	unknown	Died	
17	74	F	Bacteraemia, small-bowel obstruction	Blood	*D. desulfuricans*	None	16S rDNA	3 days	CLI, MNZ, TZP, ETP	unknown	Survived	
18	57	M	Perforated acute appendicitis	Blood	*D. desulfuricans*	In peritoneal fluid: *E. coli, K. pneumoniae,* anaerobic Gram-negative and -positive rods	16S rDNA	4 days	CLI, MNZ, SAM, ETP	unknown	Survived	
19	82	M	Bacteraemia, liver abscess	Blood	*D. desulfuricans*	None	Biochemical, 16S rDNA	15 days	AMP, AMC, IPM, PAPM, CLI, LVX	CMZ, TZP, AMC	Survived	[21]
20	73	F	Sepsis, liver abscess	Blood, pus	*D. desulfuricans*	*E. coli*	16S rDNA	3 days	LVX, MEM, SAM	MEM, SBT/CPZ, SAM, SBTPC	Survived	[22]
21	88	M	Bacteraemia, mediastinal abscess	Blood	*D. desulfuricans*	None	16S rDNA	3 days	unknown	TZP, CLDM, MNZ	Survived	[10]
22	53	M	Bacteraemia	Blood	*D. desulfuricans*	None	MALDI-TOF MS	3 days	MNZ, AMC, IPM, CLI	AMC, TZP	Survived	[23]
23	53	F	Trochanteric arthritis	Synovial fluid	*D. desulfuricans*	None	MALDI-TOF MS	6 days	MNZ, AMC	FEP, VAN, CRO, MNZ	Survived	[24]
24	67	M	Cholecystitis	Blood	*D. fairfieldensis*(*)	None	Biochemical	unknown	PEN, CLI, CHL, TET, ERY	None	Survived	[11]
25	82	M	Liver abscess	Pus	*D. fairfieldensis*	*Fusobacterium varium*	16S rDNA	7 days	MNZ	CTX, MNZ, AMP, CIP	Survived	[6]
26	75	M	Bleeding colonic polyps	Blood	*D. fairfieldensis*	None	16S rDNA	6 days	MNZ, CHL, CIP, IPM, AMC, TIM, AZM, CLI	LEX, CIP	Survived	[12]
27	46	F	Meningoencephalitis	Urine	*D. fairfieldensis*	None	16S rDNA	14 days	IPM, CIP, RIF, CLI, MNZ, CHL	AMP, RIF, EMB, INH, ACV, anti-mycobacterial drugs	Died	[5]
28	23	M	Perforating appendicitis, peritonitis	Blood	*D. fairfieldensis*	None	Biochemical, 16S rDNA	5 days	MNZ, IPM, CLI	FAM, MNZ	Survived	[3]
29	59	F	Intra-abdominal abscess	Pus	*D. fairfieldensis*	*B. vulgatus, E. lentum, E. coli, K. pneumoniae, Streptococcus intermedius*	16S rDNA	unknown	MNZ, CLI	unknown	Survived	
30	85	M	Abdominal abscess	Blood	*D. fairfieldensis*	*B. fragilis, B. uniformis, B. vulgatus, B. thetaiotaomicron, Clostridium innocuum, Clostridium* sp.*, Enterococcus avium*	16S rDNA	unknown	MNZ, CLI	unknown	Survived	
31	65	M	Abdominal wall abscess	Pus	*D. fairfieldensis*	*B. thetaiotaomicron, E. lentum, E. coli, K. pneumoniae, Proteus vulgaris, Enterococcus* sp.*, Streptococcus intermedius*	16S rDNA	unknown	MNZ, CLI	unknown	Survived	
32	32	M	Appendicitis, peritonitis	Peritoneal fluid	*D. fairfieldensis*	unknown	16S rDNA	unknown	unknown	unknown	unknown	
33	29	F	Appendicitis, peritonitis	Peritoneal fluid	*D. fairfieldensis*	unknown	16S rDNA	unknown	unknown	unknown	unknown	
34	53	F	Peritonitis	Peritoneal fluid	*D. fairfieldensis*	unknown	16S rDNA	unknown	unknown	unknown	unknown	
35	21	M	Appendicitis	Intra-abdominal collection	*D. fairfieldensis*	unknown	16S rDNA	unknown	unknown	unknown	unknown	
36–45	unknown	unknown	unknown	unknown	*D. fairfieldensis*	unknown	unknown	unknown	unknown	unknown	unknown	[4]
46	77	M	Aftercholangiopancreatography	Blood	*D. fairfieldensis*	None	16S rDNA	4 days	MNZ, CIP	TIM, CIP	Survived	[2]
47	69	F	Bacteraemia	Blood	*D. fairfieldensis*	*E. coli, Morganella morganii*	16S rDNA	9 days	MNZ, CLI, IPM, BIPM, DOR	BIPM, CFZ	Survived	[7]
48	83	M	Bacteraemia, epidural abscess	Blood	*D. fairfieldensis*	*Parvimonas micra*	MALDI-TOF MS, 16S rDNA	7 days	None	None	Survived	[25]
49	63	M	Renal cyst infection	Pus	*D. fairfieldensis*	None	16S rDNA	None	None	MEM, MNZ	Survived	This
50	64	M	Peritonitis	Peritoneal fluid	*D. piger*	unknown	16S rDNA	unknown	unknown	unknown	unknown	[14]
51	83	F	Peritonitis	Peritoneal fluid	*D. piger*	unknown	16S rDNA	unknown	unknown	unknown	unknown	
52	81	F	Rectal cancer	Peritoneal fluid	*D. piger*	unknown	16S rDNA	unknown	unknown	unknown	unknown	
53	88	F	Peritonitis	Peritoneal fluid	*D. piger*	unknown	16S rDNA	unknown	unknown	unknown	unknown	
54	14	M	Appendicitis	Abdominal collection	*D. piger*	unknown	16S rDNA	unknown	unknown	unknown	unknown	
55	18	M	Peritonitis	Peritoneal fluid	*D. piger*	unknown	16S rDNA	unknown	unknown	unknown	unknown	
56	9	M	Appendicitis, peritonitis	Peritoneal fluid	*D. piger*	unknown	16S rDNA	unknown	unknown	unknown	unknown	
57–58	unknown	unknown	unknown	unknown	*D. piger*	unknown	unknown	unknown	unknown	unknown	unknown	[4]
59	73	F	Bacteraemia, abdominal abscess	Blood	*D. piger*	*E. lenta, B. ovatus*	16S rDNA	2 days	CLI, MNZ	unknown	Survived	[20]
60	63	M	Perforated acute appendicitis	Peritoneal fluid	*D. piger*	*E. coli, Enterococcus* sp., anaerobic Gram-negative rod	16S rDNA	unknown	unknown	unknown	Survived	
61	unknown	unknown	Abdominal abscess	Peritoneal fluid	*D. vulgaris*	None	unknown	unknown	unknown	unknown	unknown	[26]
62–64	unknown	unknown	unknown	unknown	*D. vulgaris*	unknown	unknown	unknown	unknown	unknown	unknown	[4]
65	15	M	Brain abscess	Pus	*D. vulgaris*	Gram-positive cocci	Biochemical	2 days	KAN	AMC, CRO, AMK, LZD	Survived	[27]
66	70	F	Left-shoulder prosthetic-joint infection	Synovial fluid, prosthetic joint	*D. legallii*	None	16S rDNA	10 days	CLI, MNZ, ETP, AMC, CRO	unknown	Survived	[20]
67	unknown	unknown	Acute appendicitis	Peritoneal fluid	*Desulfovibrio* sp.	unknown	Biochemical	unknown	unknown	unknown	unknown	[28]
68	unknown	unknown	Perforating appendicitis	Peritoneal fluid	*Desulfovibrio* sp.	unknown	Biochemical	unknown	unknown	unknown	unknown	
69	60	M	Perforated acute appendicitis	Blood	*Desulfovibrio* sp.	*E. lenta,* anaerobic Gram-negative rod, *B. fragilis*	16S rDNA	3 days	CLI, MNZ	unknown	Survived	[20]
70	74	M	Septic shock, intra-abdominal infection	Blood	*Desulfovibrio* sp.	*Candida parapsilosis*	16S rDNA	5 days	unknown	unknown	Died	
71	45	M	Subphrenic abscess, abdominal infection	Blood	*Desulfovibrio* sp.	In subphrenic abscess: vancomycin-resistant enterococci	16S rDNA	5 days	unknown	unknown	Survived	
72	93	F	Sigmoid diverticulitis	Blood	*Desulfovibrio* sp.	None	16S rDNA	6 days	CLI, MNZ	unknown	Died	

(*) Although a human infection of *Desulfovibrio* species (specifically *D. desulfuricans,* presented as bacteraemia associated with cholecystitis) was first reported in 1987, the strain was considered as *D. fairfieldensis* in 2005

*Ref* Reference, M male, F female, *MALDI-TOF MS* Matrix-assisted laser desorption ionization time-of-flight mass spectrometry, *B* Bacteroides, *D* Desulfovibrio, *E. coli* Escherichia coli, *E. lenta* Eggerthella lenta, *E. lentum* Eubacterium lentum, *K* Klebsiella, *ACV* acyclovir, *AMC* amoxicillin-clavulanic acid, *AMK* amikacin, *AMP* ampicillin, *AMX* amoxicillin, *AZM* azithromycin, *BIPM* biapenem, *CAZ* ceftazidime, *CFZ* cefazolin, *CHL* chloramphenicol, *CIP* ciprofloxacin, *CLI* clindamycin, *CMZ* cefmetazole, *CRO* ceftriaxone, *CTX* cefpodoxime, *CXM* cefuroxime, *CZO* cefozopran, *DOR* doripenem, *DOX* doxycycline, *EMB* ethambutol, *ERY* erythromycin, *ETP* ertapenem, *FAM* cefamandole, *FEP* cefepime, *FOF* fosfomycin, *GAT* gatifloxacin, *INH* isoniazid, *IPM* imipenem, *KAN* kanamycin, *LVX* levofloxacin, *LZD* linezolid, *MEM* meropenem, *MNZ* metronidazole, *MXF* moxifloxacin, *OFX* ofloxacin, *ONZ* ornidazole, *PAPM* panipenem, *PEF* pefloxacin, *PEN* penicillin, *PIP* piperacillin, *RIF* rifampin, *SAM* ampicillin-sulbactam, *SBT/CPZ* Cefoperazone sodium and sulbactam sodium, *SBTPC* sultamicillin, *TET* tetracycline, *TIM* ticarcillin-clavulanic acid, *TZP* piperacillin-tazobactam, *VAN* vancomycin

**Table 2:** Summary of clinical characteristics of cases of infection with *Desulfovibrio* species in 27 articles

**Characteristics of cases**	
Total number of cases	72
Median age (years)	65
Female, male (%)	19, 32 (37, 63)
**Infection (%)**	
Abscess	15 (28)
Abdominal abscess	8 (15)
Liver abscess	2 (3.6)
Bacteraemia	14 (26)
Appendicitis	11 (20)
Central nervous system infection	4 (7.4)
**Source (%)**	
Blood	26 (47)
Peritoneal fluid	14 (26)
Pus	9 (16)
Urine	2 (3.6)
**Total genus/species** (%)	73
*D. fairfieldensis*	26 (36)
*D. desulfuricans*	24 (33)
*D. piger*	11 (15)
*D. vulgaris*	5 (6.8)
*D. legallii*	1 (1.4)
**Co-isolate** (%)	22 (54)
*E. coli*	9 (22)
*E. lenta (E. lentum)*	7 (17)
*K. pneumoniae*	3 (7.4)
None	19 (46)
**Identification** (%)	
16S rDNA	47 (87)
MALDI-TOF MS	3 (5.6)
Biochemical	7 (13)
**Time for positive incubation** (%)	
2 days	2 (6.9)
3 days	6 (21)
> 3 days	21 (72)
> 7 days	7 (24)
Median (days)	5
**Outcome** (%)	
Survived	34 (89)
Died	4 (11)

Percentages for each category are calculated excluding “unknown”. *D* Desulfovibrio, *E. coli* Escherichia coli, *E. lenta* Eggerthella lenta, *E. lentum* Eubacterium lentum, *K* Klebsiella, *MALDI-TOF MS* Matrix-assisted laser desorption ionization time-of-flight mass spectrometry

Because renal cyst infections in patients with ADPKD are frequent and refractory and patients on haemodialysis are immunocompromised [31], identification and eradication of the causative organism are essential [32]. The causative organisms of renal cyst infections have only been identified in 49% of cases, and the most common causative organisms are gram-negative rods from the intestinal tract [32, 33]. Therefore, the actual infection rate by *Desulfovibrio* species may be underestimated because of the difficulty in identifying anaerobic bacteria [3, 17] and the actual number of infections by anaerobic bacteria, including *Desulfovibrio* species, maybe much higher. No strain was cultured in this patient’s blood, urine, or renal cyst fluid, but *D. fairfieldensis* was detected in the renal cyst fluid by PCR testing. PCR is useful in identifying organisms that cannot be grown in vitro or in cases where existing culture techniques are not sensitive enough and/or require long incubation times due to its tremendous sensitivity, specificity, and amplification speed [34]. In previous reports, PCR tests using 16S rDNA were used to identify 87% of *Desulfovibrio* species, while biochemical methods were used in 13% (Table 2). Matrix-assisted laser desorption ionization time-of-flight mass spectrometry (MALDI-TOF MS) was also used in only 5.6% of the cases (Table 2); however, its use for organism identification is expected to increase because it is a novel method that can rapidly identify bacteria and be as accurate as 16S rDNA. In addition, 72% of the cases were identified after 3 days in cultures, and 24% were identified after 7 days (Table 2). Therefore, if the causative bacteria are unknown, performing the culture for a longer period is necessary.

In this case, contrast-enhanced CT and plain MRI identified the infected renal cyst, but 18-fluorodeoxyglucose positron emission tomography/CT (18FDG PET/CT) has been reported to be useful in the diagnosis of renal cyst infection [35, 36]. However, this method is not commonly used in Japan due to cost, where the national health insurance system allows the use of 18FDG PET/CT for malignant tumours mainly.

The routes of renal cyst infection include hematogenous routes and retrograde infection from the urinary tract. In the literature review, bloodstream infection was the most common among *Desulfovibrio* infection, followed by intra-abdominal infection, while urinary tract infection was less common at 3.6% (Table 2). He was in regular contact with soil and sewage, which are dwelling sites of the bacteria, due to his occupation. Since most of the *Desulfovibrio* species are also found in the environment, and since haemodialysis patients have reduced urine volume and are unable to cleanse themselves through urination, we suspected that the bacteria had entered the urinary tract and caused the infection retrogradely. However, it has been reported that *D. fairfieldensis* survives only in the human intestinal tract [4, 25], and we thought that it was more likely that the infection was haematogenous.

Infected cysts need early percutaneous cyst drainage, which provides the best treatment results because antibiotics alone do not usually treat the infection [33, 37]. In this case, the patient’s condition improved after drainage was performed.

For antimicrobial treatment of renal cyst infections, lipid-permeable antimicrobials with high penetration are recommended as first-line agents [32, 37]. Therefore, we also used ciprofloxacin as a quinolone, but with poor improvement. Then, we used meropenem which has been reported to have clinical improvement for cyst infection despite the poor penetration [8], but there was no improvement. The other antimicrobial agents for this patient were used as empirical treatments.

Optimal antimicrobial therapy for *D. fairfieldensis* remains controversial. One study showed that metronidazole had the highest antibacterial activity, while imipenem was effective against it [1]. Another study showed that imipenem, ciprofloxacin, clindamycin, chloramphenicol, and beta-lactams, except carbapenems, were ineffective [2]. Lipid-permeable antimicrobials such as metronidazole and clindamycin increase the concentrations of the antimicrobials in the renal cyst fluid [38]. Therefore, oral metronidazole was used for this patient. In addition, *D. fairfieldensis* may be more resistant to antimicrobial agents and have higher pathogenicity than other *Desulfovibrio* species [1–3]. Metronidazole was effective with good blood levels in the renal cysts of patients with ADPKD, including those on haemodialysis [38]. Summarising the previous reports of *Desulfovibrio* species infection, metronidazole showed the highest susceptibility (78%), and clindamycin was also effective (Table 3). However, metronidazole was used in only 23% of the patients; given that *D. fairfieldensis* is more resistant to antimicrobial agents and more pathogenic than other *Desulfovibrio* species [1–4], identifying the *Desulfovibrio* species, especially in *D. fairfieldensis*, by PCR tests, and using metronidazole, are essential for patient prognosis. In addition, because 54% of the patients with *Desulfovibrio* infection were complicated with other bacteria, there is concern that *Desulfovibrio* species can manifest when antimicrobial agents which are susceptible to other bacteria but resistant to *Desulfovibrio* are used (Table 2). The prognosis of *Desulfovibrio* infection was 11% of death, and treatment should be carefully selected, including appropriate drainage and antimicrobial agents.

**Table 3:** Summary of antimicrobial susceptibility of *Desulfovibrio* species and actual antibiotic therapy

	Antimicrobial susceptibility (%)	Antimicrobial therapy (%)
MNZ	25 (78)	5 (23)
CLI	21 (66)	1 (4.5)
IPM	10 (31)	1 (4.5)
AMC	9 (28)	3 (14)
ETP	4 (13)	0
PEN	3 (9.4)	0
SAM	3 (9.4)	2 (9.1)
MEM	3 (9.4)	2 (9.1)
LVX	3 (9.4)	0
CIP	3 (9.4)	3 (14)
TZP	2 (6.3)	4 (18)
PAPM	1 (3.1)	0
AMP	1 (3.1)	2 (9.1)
CRO	1 (3.1)	3 (14)

*AMC* amoxicillin-clavulanic acid, *AMP* ampicillin, *CIP* ciprofloxacin, *CLI* clindamycin, *CRO* ceftriaxone, *ETP* ertapenem, *IPM* imipenem, *LVX* levofloxacin, *MEM* meropenem, *MNZ* metronidazole, *PAPM* panipenem, *PEN* penicillin, *SAM* ampicillin-sulbactam, *TZP* piperacillin-tazobactam

The essential recommendations for the general treatment of renal cyst infection, including *Desulfovibrio* species, are as follows: if the bacteria of renal cyst infection are unknown, focus on long-term culture studies, consider identification of the organism by 16S rDNA or MALDI-TOF MS, consider the possibility of multiple bacterial complications. Some bacteria have a high mortality rate, and drainage should be performed first if possible and appropriate antimicrobials should be administered according to the organism.

To conclude, this is the first report of a renal cyst infection with the genus *Desulfovibrio* species to the best of our knowledge. *D. fairfieldensis* has higher pathogenicity and more antimicrobial resistance than other *Desulfovibrio* species and is difficult to detect. PCR tests can detect this bacterium and ensure better management for a successful recovery.

## Data Availability

All data generated or analysed during this study are included in the published article.

## Abbreviations

ADPKD: Autosomal dominant polycystic kidney disease
CRP: C-reactive protein
CT: Computed tomography
MRI: Magnetic resonance imaging
PCR: Polymerase chain reaction
18FDG PET/CT: 18-fluorodeoxyglucose positron emission tomography/computed tomography
MALDI-TOF MS: Matrix-assisted laser desorption ionization time-of-flight mass spectrometry
